# Anxiety, depression, and their comorbidity among Chinese college students during the COVID-19 lockdown in the post-epidemic era: an online cross-sectional survey

**DOI:** 10.1186/s12888-023-05442-z

**Published:** 2023-12-08

**Authors:** Jinghong Huang, Xiaojun Liu

**Affiliations:** 1 School of Health Management, Fujian Medical University, Fuzhou, China; 2 School of Public Health, Lanzhou University, Lanzhou, China

**Keywords:** COVID-19, Chinese college students, Anxiety, Depression, Comorbidity, Lockdown

## Abstract

**Background:**

The coronavirus disease 2019 (COVID-19) pandemic continues to affect the mental health of college students in the post-epidemic era. We assessed the status and related factors of college students who are vulnerable to anxiety and depression during the COVID-19 lockdown.

**Methods:**

This cross-sectional study was conducted two weeks after the beginning of the COVID-19 lockdown, from November 6, 2022, to December 2, 2022, with 1176 valid samples using convenience sampling. The Generalized Anxiety Disorder-7 (GAD-7) and Patient Health Questionnaire-9 (PHQ-9) were used to measure levels of anxiety and depression in college students. Factors related to anxiety, depression, and their comorbidity were analyzed using binary logistic regression.

**Results:**

The prevalence of anxiety, depression, and their comorbidity were 27.04%, 34.10%, and 25.60%, respectively. There was a higher risk of anxiety, depression, and their comorbidity among those who were currently living in rural areas. Compared with individuals with low monthly living expenses, those with higher monthly living expenses were less prone to anxiety, depression, and their comorbidity.

**Conclusions:**

High prevalence of anxiety, depression, and their comorbidity were detected among college students during the COVID-19 lockdown. These were most common among college students who were senior students, had abnormal body mass index (BMI), were rural area residents, did study arts and humanities, were one-child in the family, and had low monthly living expenses and poor academic performance. Intervention practitioners and policymakers should formulate individualized prevention and intervention measures during the COVID-19 lockdown in the post-epidemic and possible future pandemics for college students.

## Background

Mental disorders have been gradually showing younger ages of onset, with anxiety and depression being the most common [1], and they share many risk factors and are often comorbid [2]. However, this global health crisis continues to be overlooked, especially in the context of COVID-19 pandemic [1]. Coronavirus disease 2019 (COVID-19) is an acute respiratory infection that emerged in 2019. As a public health emergency, it has spread worldwide, resulting in a global health crisis and pandemic [3]. Many countries and regions gradually restricted border crossings and social activities to control the spread of the disease [4]. Previous studies have confirmed increases in the prevalence of anxiety and depression during COVID-19 outbreak. A meta-analysis of mental health in college students during the COVID-19 pandemic showed that the prevalence of anxiety and depression were 39% and 36%, respectively, much higher than before the COVID-19 outbreak [5], particularly among young adults [6].

As it has now been almost three years since the COVID-19 outbreak, and the threat to the global population has diminished significantly, many countries have reduced their implemented restrictions, with the world gradually entering the post-epidemic era. However, some social isolation measures are still in place (e.g., in education). By the end of 2022, most universities in China continued to restrict social gatherings and free movement of students and staff to prevent large-scale cross-infection. More specifically, the spread of COVID-19 was prevented by limiting the access of staff and students to the college. Increases in such stressful events can predict increases in mental disorder prevalence [7]. Importantly, the changes in lifestyle and social distancing brought about by lockdown can influence the mental health status of individuals, with college students being an especially vulnerable population [8]. Although lockdowns are different from social epidemic control, they cause high levels of social isolation, preventing college students from obtaining social support and affecting their physical and mental health [9, 10]. Surveyed young adults stated that they feared the consequences of COVID-19 in terms of families suffering from disease, death, unemployment, economic hardship, and academic regression [10].

There have been many studies on the mental health of college students under lockdowns. Evans et al. suggested that levels of depression significantly increased because of lockdowns [11]. A large epidemiological study in college students in China showed a higher prevalence of mental disorders in the early stages of the COVID-19 outbreak than once restrictions and lockdown were relaxed [12]. Indeed, the psychological problems caused in the early stages of COVID-19 stemmed from a lack of understanding of the virus [13]. As individuals adapted to the epidemic management strategies, the prevalence of mental disorders decreased [14]. However, after multiple rounds of outbreaks, the cascading preventive and control measures have had an increasingly negative impact on the mental health of students, continuing to exist even in the post-epidemic era [15].

According to the latest statistical bulletin on education development in China, there are 44.3 million students enrolled in higher education. However, research on the effects of COVID-19 on mental health has focused on the early stages of the pandemic [16, 17], and there are few reports on the effects of lockdown and social restrictions on the mental health of college students in the post-epidemic era [14]. Therefore, this study investigated the mental health status of college students under COVID-19 lockdown in the post-epidemic era, analyzed related factors, and provided a scientific basis for control strategies for any future pandemics.

## Methods

### Study design and participants

Participants were recruited from Chinese college students through an online questionnaire from November 6, 2022, to December 2, 2022, using convenience sampling. Due to the restricted conditions of the study (COVID-19 lockdown management), the online survey link created by QuestionnaireStar was distributed via QQ and WeChat to any Chinese college students who have been under COVID-19 lockdown management for a minimum of two weeks. Questionnaire Star is a widely used online survey platform in China. WeChat and QQ are commonly used chat software among Chinese. The questionnaire collected questions about social demographic characteristics and mental health status. The questions of mental health status mostly referred to two weeks after the beginning of the COVID-19 lockdown. The following participants were included: (i) those who were college students in China, (ii) those who have been under COVID-19 lockdown management for a minimum of two weeks. Consequently, we received 1188 online questionnaires. In order to ensure data accuracy, the logic of the questionnaire was checked after completing the survey. There were 12 questionnaires were excluded, and the effective rate of the response was 98.99%. Hence, a total of 1176 valid samples were included in the study.

### Measures

Social demographic characteristics: gender (male and female), grade was recorded as freshman, sophomore, junior, and senior (including fourth and fifth-year college and graduate students) according to the stage the participant was actually attending, body mass index (BMI) was calculated by weight (kg) divided by height (m^2^), and further divided into the following three groups: underweight (BMI < 18.50), normal (BMI = 18.50-23.99), and overweight (BMI ≥ 24) [18], current residence (rural and urban), major was classified as arts and humanities, science and technology, medicine, and others (including agronomy and other majors), being one-child in the family (yes and no), monthly living expenses was classified as low (≤ 999 CNY), middle (1000–1499 CNY), high (1500–1999 CNY), and super (≥ 2000 CNY), source of monthly living expenses (family and others), family structure (two-parent families and others), and academic performance ranking was classified as 0-25%, 25.10-50%, 50.10-75%, and 75.10-100%.

Mental health status: The Generalized Anxiety Disorder-7 (GAD-7) was a self-report measure of anxiety. The scale consists of seven questions, each was scored four level (0 = none, 1 = few days, 2 = most of the time, and 3 = almost every day). The total score ranging from 0 to 21. The Patient Health Questionnaire-9 (PHQ-9) was a self-report measure of depression, assessing severity through nine queastions. Each item was scored as: 0 = none, 1 = few days, 2 = most of the time, and 3 = almost every day. The total score ranging from 0 to 27. Anxiety and depression positively correlated with their scores. The GAD-7 and PHQ-9 have been authenticated to be excellent reliable and valid [19, 20]. More than 10 points mean anxiety [20] and depression [19]. The comorbidity was positive when both anxiety and depression were detected [2].

### Statistical analyses

Firstly, the frequencies and proportions were used to describe the social demographic characteristics. Secondly, the Chi-square tests were used to explore whether differed between mental disorders and social demographic characteristics. Moreover, related factors of mental health in Chinese college students were obtained from the binary logistic regression analysis. Anxiety, depression, and their comorbidity as dependent variables, respectively. Gender, grade, BMI, current residence, major, being one-child in the family, monthly living expenses, source of monthly living expenses, family structure and academic performance ranking were included in the model as independent variables. Statistical analyses were conducted using Stata statistical software (version 17.0). *P* < 0.05 was set as statistical significance.

## Results

### Social demographic characteristics

**Fig. 1:**
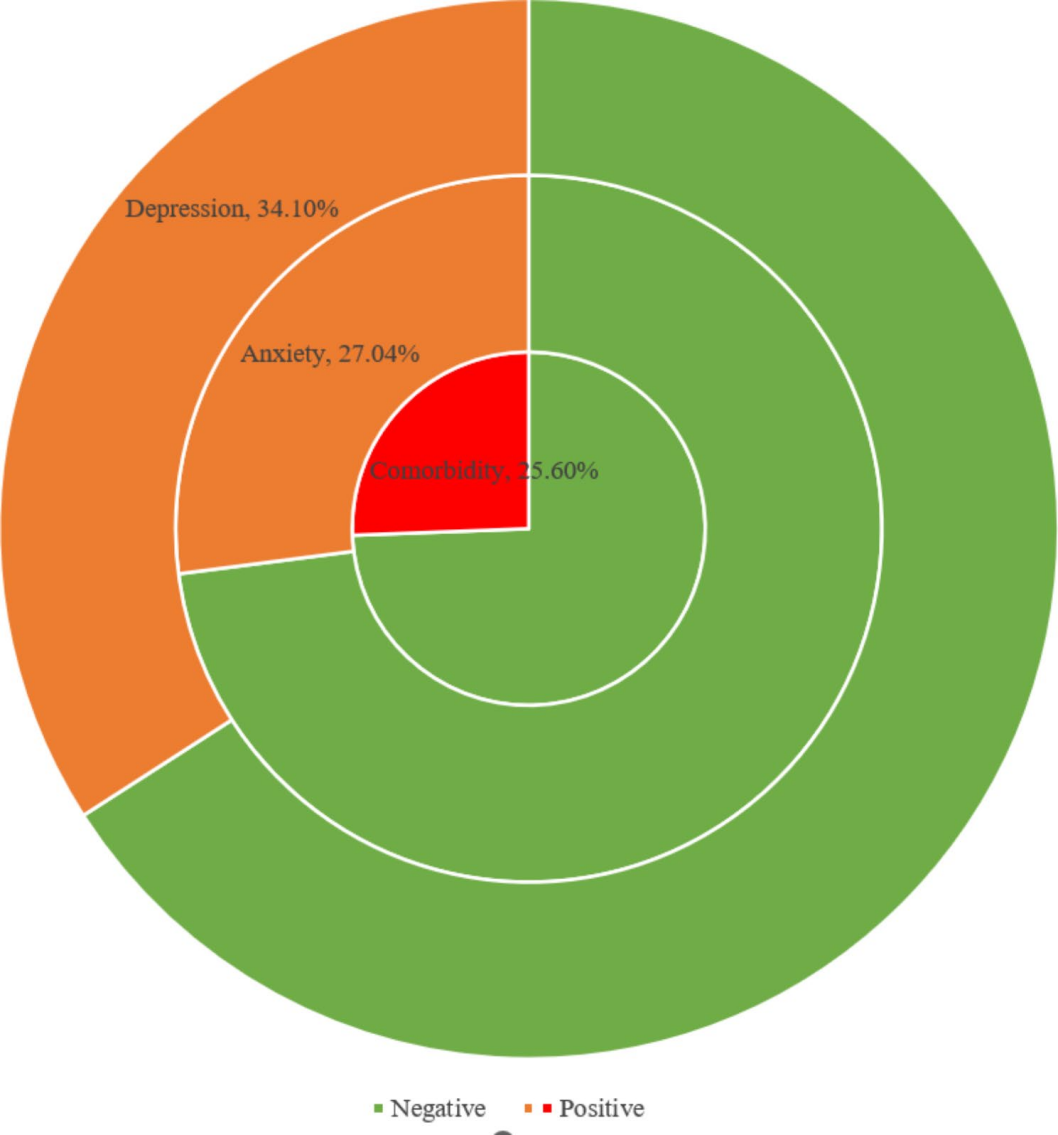
Prevalence of anxiety, depression, and their comorbidity

A total of 1176 participants were included in this study, with a minimum age of 15 years, a maximum age of 29 years, and a median age of 19 years, with the majority of participants being 18 years old. Participants were more likely to be female, freshman, normal BMI, currently living in urban areas, majored in management, non one-child in their families, have high monthly living expenses, monthly living expenses from family, two-parent families, academic performance ranked 25.10-50%. The detailed results of social demographic characteristics are shown in Table 1. As illustrated in Fig. 1, the prevalence of anxiety, depression, and their comorbidity were 27.04%, 34.10%, and 25.60%.

**Table 1:** Basic information of participants in the survey

Characteristics	Categories	Frequency	Proportion (%)
**Gender**	Male	533	45.32
	Female	643	54.68
**Grade**	Freshman	458	38.95
	Sophomore	229	19.47
	Junior	214	18.20
	Senior	275	23.38
**BMI**	Underweight	207	17.60
	Normal	783	66.58
	Overweight	186	15.82
**Current residence**	Rural	584	49.66
	Urban	592	50.34
**Major**	Arts and Humanities	214	18.20
	Science and Technology	297	25.26
	Medicine	205	17.43
	Management	384	32.65
	Others	76	6.46
**Being one-child in the family**	Yes	438	37.24
	No	738	62.76
**Monthly living expenses**	Low	148	12.59
	Middle	352	29.93
	High	401	34.10
	Super	275	23.38
**Source of monthly living expenses**	Family	910	77.38
	Others	266	22.62
**Family structure**	Two-parent families	1066	90.65
	Others	110	9.35
**Academic performance ranking**	0-25%	296	25.17
	25.10-50%	337	28.66
	50.10-75%	325	27.64
	75.10-100%	218	18.54

Abbreviation: BMI: body mass index

### Distribution of mental disorders in college students with different characteristics

As shown in Table 2, gender, grade, BMI, current residence, major, being one-child in the family, monthly living expenses, source of monthly living expenses, family structure, and academic performance ranking differed across whether had anxiety, depression, and their comorbidity (all *P* < 0.05).

**Table 2:** Distribution of anxiety, depression, and their comorbidity with different characteristics

Characteristics	Categories	Anxiety	Depression	Comorbidity
		Positive	Positive	Positive
**Gender**		(16.557, *P* < 0.001)	(6.898, *P* = 0.009)	(20.314, *P* < 0.001)
	Male	175 (32.83)	203 (38.09)	170 (31.89)
	Female	143 (22.24)	203 (30.79)	131 (20.37)
**Grade**		(41.183, *P* < 0.001)	(37.591, *P* < 0.001)	(49.489, *P* < 0.001)
	Freshman	84 (18.34)	112 (24.45)	74 (16.16)
	Sophomore	61 (26.64)	81 (35.37)	58 (25.33)
	Junior	88 (41.12)	100 (46.73)	87 (40.65)
	Senior	85 (30.91)	108 (39.27)	82 (29.82)
**BMI**		(17.196, *P* < 0.001)	(19.647, *P* < 0.001)	(18.782, *P* < 0.001)
	Underweight	62 (29.95)	75 (36.23)	61 (29.47)
	Normal	185 (23.63)	238 (30.40)	172 (21.97)
	Overweight	71 (38.17)	88 (47.31)	68 (36.56)
**Current residence**		(32.001, *P* < 0.001)	(19.470, *P* < 0.001)	(29.331, *P* < 0.001)
	Rural	201 (34.42)	235 (40.24)	190 (32.53)
	Urban	117 (19.76)	166 (28.04)	111 (18.75)
**Major**		(81.595, *P* < 0.001)	(82.991, *P* < 0.001)	(87.835, *P* < 0.001)
	Arts and Humanities	110 (51.40)	129 (60.28)	108 (50.47)
	Science and Technology	70 (23.57)	91 (30.64)	66 (22.22)
	Medicine	49 (23.90)	64 (31.22)	45 (21.95)
	Management	77 (20.05)	98 (25.52)	71 (18.49)
	Others	12 (15.79)	19 (25.00)	11 (14.47)
**Being one-child in the family**		(22.027, *P* < 0.001)	(15.207, *P* < 0.001)	(26.002, *P* < 0.001)
	Yes	153 (34.93)	180 (41.10)	149 (34.02)
	No	165 (22.36)	221 (29.95)	152 (20.60)
**Monthly living expenses**		(145.754, *P* < 0.001)	(103.979, *P* < 0.001)	(151.233, *P* < 0.001)
	Low	100 (65.57)	104 (70.27)	98 (66.22)
	Middle	85 (24.15)	114 (32.39)	80 (22.73)
	High	70 (17.46)	99 (24.69)	65 (16.21)
	Super	63 (22.91)	84 (30.55)	58 (21.09)
**Source of monthly living expenses**		(26.933, *P* < 0.001)	(13.836, *P* < 0.001)	(25.987, *P* < 0.001)
	Family	213 (23.41)	285 (31.32)	201 (22.09)
	Others	105 (39.47)	116 (43.61)	100 (37.59)
**Family structure**		(5.346, *P* = 0.021)	(15.261, *P* < 0.001)	(7.389, *P* = 0.007)
	Two-parent families	278 (26.08)	345 (32.26)	261 (24.48)
	Others	40 (36.36)	56 (50.91)	40 (36.36)
**Academic performance ranking**		(21.732, *P* < 0.001)	(22.920, *P* < 0.001)	(21.808, *P* < 0.001)
	0-25%	65 (21.96)	75 (25.34)	59 (19.93)
	25.10-50%	77 (22.85)	109 (32.34)	74 (21.96)
	50.10-75%	92 (28.31)	119 (36.62)	88 (27.08)
	75.10-100%	84 (38.53)	98 (44.95)	80 (36.70)

### Related factors mental health of college students

The senior college students had a higher risk of anxiety, depression, and their comorbidity than the freshman. The participants who were overweight were at higher risk of suffering from depression, and the underweight had a higher likelihood of having comorbidity than those who had a normal BMI. The individuals who were currently living in rural areas had more chance of having anxiety, depression, and their comorbidity. The college students who did not study arts and humanities had less chance of suffering from anxiety, depression, and their comorbidity. Those who were one-child were more likely to experience anxiety and comorbidity. Moreover, monthly living expenses are a significant factor relating mental health; compared with the participants who had low monthly living expenses, those who had higher monthly living expenses were less prone to anxiety, depression, and their comorbidity. The participants in other family structures felt more depression than those in two-parent families. The participants whose monthly living expenses were from other approaches were more likely to suffer from anxiety and comorbidity than those whose monthly living expenses came from family. We also found that those who ranked low academic performance were more likely to suffer from depression than the participants who ranked 0-25%. All binary logistic regression analyses among different mental health disorders were summarized in Table 3.

**Table 3:** Binary logistic regression analysis of relating factors on mental health

Characteristics	Categories	Anxiety			Depression			Comorbidity		
		OR	95% CI	*P*	OR	95% CI	*P*	OR	95% CI	*P*
**Gender (reference =** Female)	Male	1.23	0.89–1.71	0.211	1.04	0.77–1.41	0.795	1.33	0.95–1.86	0.098
**Grade (reference =** Freshman)	Sophomore	1.63	1.08–2.46	**0.021**	1.79	1.23–2.61	**0.002**	1.79	1.17–2.74	**0.007**
	Junior	1.56	1.00-2.46	0.056	1.79	1.17–2.73	**0.007**	1.77	1.11–2.82	**0.016**
	Senior	1.66	1.11–2.48	**0.013**	1.92	1.33–2.77	**< 0.001**	1.87	1.24–2.83	**0.003**
**BMI (reference =** Normal**)**	Underwieght	1.42	0.96–2.09	0.078	1.25	0.88–1.80	0.217	1.57	1.05–2.33	**0.026**
	Overweight	1.27	0.85–1.91	0.244	1.49	1.02–2.16	**0.038**	1.27	0.84–1.93	0.264
**Current residence (reference =** Urban)	Rural	1.82	1.31–2.54	**< 0.001**	1.42	1.05–1.91	**0.023**	1.74	1.24–2.44	**0.001**
**Major (reference =** Arts and Humanities)	Science and Technology	0.46	0.29–0.71	**0.001**	0.45	0.29–0.68	**< 0.001**	0.44	0.28–0.69	**< 0.001**
	Medicine	0.49	0.31–0.79	**0.003**	0.45	0.29–0.70	**< 0.001**	0.47	0.29–0.76	**0.002**
	Management	0.39	0.25–0.60	**< 0.001**	0.34	0.22–0.50	**< 0.001**	0.37	0.24–0.57	**< 0.001**
	Others	0.25	0.12–0.52	**< 0.001**	0.31	0.17–0.59	**< 0.001**	0.24	0.11–0.51	**< 0.001**
**Being one-child in the family (reference =** No)	Yes	1.51	1.08–2.11	**0.015**	1.29	0.95–1.76	0.098	1.54	1.10–2.16	**0.013**
**Monthly living expenses (reference =** Low)	Middle	0.25	0.15–0.40	**< 0.001**	0.32	0.20–0.51	**< 0.001**	0.24	0.15–0.40	**< 0.001**
	High	0.19	0.12–0.32	**< 0.001**	0.25	0.15–0.40	**< 0.001**	0.19	0.11–0.31	**< 0.001**
	Super	0.29	0.17–0.50	**< 0.001**	0.35	0.21–0.58	**< 0.001**	0.28	0.16–0.47	**< 0.001**
**Source of monthly living expenses (reference =** Family**)**	Others	1.64	1.17–2.31	**0.004**	1.23	0.89–1.70	0.217	1.56	1.10–2.21	**0.012**
**Family structure (reference =** Two-parent families)	Others	1.14	0.70–1.85	0.593	1.75	1.13–2.72	**0.013**	1.28	0.78–2.07	0.327
**Academic performance ranking (reference =** 0-25%)	25.10-50%	1.04	0.69–1.56	0.844	1.46	1.01–2.12	**0.047**	1.14	0.75–1.72	0.552
	50.10-75%	1.09	0.72–1.66	0.165	1.49	1.01–2.19	**0.043**	1.17	0.76–1.80	0.480
	75.10-100%	1.38	0.88–2.16	0.084	1.69	1.11–2.58	**0.015**	1.38	0.87–2.21	0.171

## Discussion

The current study evaluated the mental health of college students during a COVID-19 lockdown in the post-epidemic era. The results indicated that anxiety, depression, and their comorbidity remained at high levels, and the students’ mental health status was concerning. In a meta-analysis combining 27 relevant studies (706,415 total participants), the prevalence of anxiety and depression in college students were lower than at the beginning of the COVID-19 pandemic from December 2019 to October 2020 (anxiety: 36.00% vs. 27.04%, depression: 39.00% vs. 34.10%) [5]. The early stages of the COVID-19 pandemic led to a rapid spread of the disease and psychological problems, especially among college students, due to the highly contagious and pathogenic nature of the virus and inadequate treatment programs [4]. As the COVID-19 outbreak prevention and control measures were gradually adapted, the concerns of college students about their families and their situations likely decreased [15]. Still, the possibility of an increase in mental disorders due to psychological stress and trauma cannot be ruled out [12]. Additionally, a systematic review and meta-analysis revealed that college students in China had lower rates of anxiety and depression than students in other regions of the world, including Africa, South America, North America, and Oceania [14]. This is likely the result of gradual improvements in the Chinese public health system in recent years, strict COVID-19 prevention and control policies, and a high level of cooperation of the public in China [21].

Academic and employment pressure is a common concern for college students and may affect their mental health [14]. Studies have indicated that low-achieving students are more likely to receive negative feedback from schools and parents, which increases their risk of mental disorders [10, 22]. Previous studies have shown that, under the influence of major social events, students are prone to poor concentration and cognitive decline, increasing the risk of depression and affecting their academic performance and employment [10, 23]. In addition, individuals with depression tend to develop negative emotions such as underestimating themselves in the face of schoolwork and future employment, thus creating a vicious cycle of academic decline and depression [24], which supports the conclusions of the present study that poor academic performance was associated with a higher prevalence of depression among senior college students. However, in the present study, the statistical association between academic performance and mental disorders was only present for depression, but this may be a consequence of the sample size [25]. Moreover, the chosen study program also affects mental health in college students [26]; those who did not study arts and humanities generally experience less academic stress academically and, therefore, less psychological stress caused by their studies [14].

The present study revealed that a body mass index outside of the range considered “healthy” was a risk factor for depression and comorbid anxiety and depression in college students, which was consistent with prior studies [24, 27]. High or low BMI can somewhat cause social stress for oneself and influence mental health [28]. Moreover, under COVID-19 lockdown management, reduced opportunities for physical activity and socialization and possibly increased poor dietary behaviors may accelerate the BMI beyond the “healthy” range and the development of mental disorders [29, 30]. Previous studies have demonstrated that family financial support has an important influence on the mental health of college students [31], which supports the finding that higher monthly living expenses were a protective factor for mental disorders in the present study. In students who are financially independent, low and/or unstable levels of financial support can cause academic and financial pressure [32]. Furthermore, social and environmental factors, such as social support, can also affect mental health [33, 34]. Under China’s long-term dualistic urban-rural structure, there has been a significant difference in the distribution of social resources between urban and rural areas, leading to higher socioeconomic stresses in families from rural areas [35, 36].

Generally, children without siblings receive better care and support from their parents in a good family function and are, therefore, at a lower risk of developing mental disorders than children with siblings [37]. Interestingly, however, the present study found that students without siblings were more likely to experience mental disorders, this was consistent with Cheng et al.’s findings [38]. The potential mechanism for this may be that children in one-child families in China tend to face particularly higher expectations from their parents [38]. Child tend to receive more negative feedback and impaired emotional expression when family functioning is poor or at higher levels of dysfunction, and without the buffering effect of sibling interactions, thus increasing the risk of developing mental disorders [39–41]. This inference is supported by the fact that the proportion of single-parent families among the children without siblings in the present study was not low (42.73%). Moreover, the present study showed that college students in families without two parents were at a higher risk for mental disorders.

In addition, compared to general population during the same period, college students are at higher risk for mental disorders [42, 43]. This may be attributed to college students having lower financial independence and being more susceptible to family support and stressful events [33]. Meanwhile, the finding that women are more likely to have mental disorders may be limited in its generalization to the college student due to the relatively lower social pressures on them [44]. Overall, the impact of lockdown management on the mental health of college students is relatively specific and requires the development of individualized prevention and intervention measures.

The results of this study have important significance for identifying college students who may be at risk of anxiety, depression, and their comorbidity in the COVID-19 lockdown management in the post-epidemic as well as possible future major public health events. However, this study had several limitations. First, as a consequence of the COVID-19 pandemic, this study used online convenience sampling, which may have caused sampling bias. Second, mental disorders are significantly heterogeneous in the college student population, and subgroups were not compared. Third, this study research was based on a cross-sectional study, which needs to be revised to reveal causal relationships. Finally, more potential confounders were not controlled, such as the previous history of mental disorders, physical activity, and dietary behaviors. Future studies may consider the above limitations.

## Conclusions

The present study evaluated the prevalence and related factors of mental health among college students under the COVID-19 lockdown late in the epidemic, and found that the high prevalence of anxiety, depression, and their comorbidity were found among college students in China. Our results suggest that in the post-epidemic era and in possible future pandemics, attention should be given to senior, abnormal BMI, students from rural areas, those studying subjects of arts and humanities, children without siblings, and students with low monthly living expenses and poor academic performance. Intervention practitioners and policymakers need to establish effective screening procedures, which contribute to identifying students who are at high risk for mental health problems during large-scale stressful events (e.g., major public health events such as COVID-19 lockdown management) and effectively intervene to prevent serious consequences.

## Data Availability

This can be obtained by contacting the corresponding author.

## Abbreviations

COVID-19: Coronavirus disease 2019
GAD-7: Generalized Anxiety Disorder-7
PHQ-9: Patient Health Questionnaire-9
BMI: Body mass index
OR: Odds ratio
CI: Confidence interval
